# Simulated Microgravity Affects Pro-Resolving Properties of Primary Human Monocytes

**DOI:** 10.3390/cells13010100

**Published:** 2024-01-03

**Authors:** Alessandro Leuti, Marina Fava, Niccolò Pellegrini, Giulia Forte, Federico Fanti, Francesco Della Valle, Noemi De Dominicis, Manuel Sergi, Mauro Maccarrone

**Affiliations:** 1 Department of Medicine, Campus Bio-Medico University of Rome, 00128 Rome, Italy; n.pellegrini@unicampus.it (N.P.); giuforte99@gmail.com (G.F.); 2 European Center for Brain Research, IRCCS Santa Lucia Foundation, 00143 Rome, Italy; m.fava@unicampus.it; 3 Department of Bioscience and Technology for Food, Agriculture and Environment, University of Teramo, 64100 Teramo, Italy; ffanti@unite.it (F.F.); fdellavalle742@gmail.com (F.D.V.); 4 Department of Physics, University of Trento, 38123 Trento, Italy; noemi.dedominicis@student.univaq.it; 5 Department of Biotechnological and Applied Clinical and Sciences, University of L’Aquila, 67100 L’Aquila, Italy; 6 Department of Chemistry, Sapienza University of Rome, 00185 Rome, Italy; manuel.sergi@uniroma1.it

**Keywords:** microgravity, resolution of inflammation, monocytes, specialized pro-resolving mediators, resolvins

## Abstract

Space-related stressors such as microgravity are associated with cellular and molecular alterations of the immune and inflammatory homeostasis that have been linked to the disorders that astronauts suffer from during their missions. Most of the research of the past 30 years has consistently established that innate adaptive immune cells represent a target of microgravity, which leads to their defective or dysfunctional activation, as well as to an altered ability to produce soluble mediators—e.g., cytokines/chemokines and bioactive lipids—that altogether control tissue homeostasis. Bioactive lipids include a vast array of endogenous molecules of immune origin that control the induction, intensity and outcome of the inflammatory events. However, none of the papers published so far focus on a newly characterized class of lipid mediators called specialized pro-resolving mediators (SPMs), which orchestrate the “resolution of inflammation”—i.e., the active control and confinement of the inflammatory torrent mostly driven by eicosanoids. SPMs are emerging as crucial players in those processes that avoid acute inflammation to degenerate into a chronic event. Given that SPMs, along with their metabolism and signaling, are being increasingly linked to many inflammatory disorders, their study seems of the outmost importance in the research of pathological processes involved in space-related diseases, also with the perspective of developing therapeutic countermeasures. Here, we show that microgravity, simulated in the rotary cell culture system (RCCS) developed by NASA, rearranges SPM receptors both at the gene and protein level, in human monocytes but not in lymphocytes. Moreover, RCCS treatment reduces the biosynthesis of a prominent SPM like resolvin (Rv) D1. These findings strongly suggest that not only microgravity can impair the functioning of immune cells at the level of bioactive lipids directly involved in proper inflammation, but it does so in a cell-specific manner, possibly perturbing immune homeostasis with monocytes being primary targets.

## 1. Introduction

The ability of microgravity to rearrange cellular and molecular properties of immune cells has been proposed as a main mechanism responsible for several alterations observed in astronauts experiencing weightlessness, since the very first Apollo and Skylab missions [1,2].

These disorders, which have been thoroughly discussed in the past 50 years, include loss of bone and muscle mass [3], enhanced sensitivity to pathogens [1,4], inflammatory conditions [5] and neurological consequences [6]. Such pathophysiological alterations can be accompanied—and are partly thought to be caused—by a direct effect of microgravity on bone and muscle cells, on the release of hormones and, relevant for this work, on immune cells [7]: indeed, many authors reported that authentic or simulated weightlessness can act on lymphocytes, leading to a reduced CD3/CD28-based activation and replication, altered cytokine release pattern and increased apoptosis [8]. Microgravity also affects macrophages, with diverse effects on migration, the expression of adhesion molecules, the production of immunomodulatory factors and the expression of M2 macrophage-like markers [9]. By contrast, scarce data are available on the effects of microgravity on circulating monocytes. Among the many functions of immune cells that are targeted by microgravity, the production of soluble immunomodulatory mediators seems crucial. In particular, these cells are able to produce a vast array of pro- and anti-inflammatory cytokines, as well as a plethora of other endogenous compounds of lipid nature that are the backbone of the immune response, its induction and its regulation. Although the effects of microgravity on the production of bioactive lipids has been addressed to some degree in the past decades, with the entirety of the efforts being focused on eicosanoids and, to some extent, endocannabinoids [4], the vast majority of other pivotal bioactive lipids involved in tissue homeostasis remains an uncharted territory in space biology. Specialized pro-resolving mediators (SPM) are a recently characterized family of immunomodulatory molecules that are produced by monocytes/macrophages, neutrophils, platelets and hypoxic endothelia from arachidonic, docosahexaenoic, docosapentaenoic and eicosapentaenoic acids (AA, DHA, DPA and EPA, respectively) through the action of 5-, 12- and 15-lipoxygenase (5-, 12- and 15-LOX); of acetylated cyclooxygenase-2 (COX-2); and of cytochrome P450. The main role of SPMs is the reduction in neutrophil influx at the inflamed site, the inactivation of pro-inflammatory immune cells and the clearance of tissue debris, in a process called “resolution of inflammation” that is meant to avoid irreversible tissue damage coming from chronic inflammation [10,11]. SPMs include more than 40 compounds, belonging to five main classes of lipids, namely D-series and E-series resolvins (RvD and RvE), maresins (MaR), protectins and lipoxins (LX), with only a handful of their target receptors being known to date, including GPR32/18/101, formyl peptide receptor 2 (FPR2, also known as ALX), chemerin receptor 23 (ChemR23) and Leucine-rich repeat-containing G-protein coupled receptor 6 (LGR6) [11]. Of note, the dysfunction of their signaling or metabolism has recently been linked to many known diseases—including arthritis, diabetes, Parkinson’s and Alzheimer’s disease and multiple sclerosis—and virtually implied in any condition that features inflammation either as a primary pathogenic mechanism or as a collateral consequence [11]. Microgravity has consistently been associated with inflammatory alterations of the immune cells and of their products, both in vivo and in several in vitro cellular models, suggesting that this space-related stressor alters the entire machinery of immune homeostasis. Nevertheless, the effect of microgravity on the resolution system, and on the cells that produce SPMs or represent their target, remains completely unknown to date. In the present work we investigated, for the first time, the effect of microgravity—simulated by means of the rotary cell culture syst em (RCCS) developed by NASA—on the resolution system of primary monocytes and lymphocytes isolated from healthy donors.

We show that simulated microgravity can alter the expression of GPR32 and GPR18—two prominent SPM receptors—as well as the biosynthesis of one of their endogenous ligand (RvD1) but not of another (RvD2). Furthermore, we show that simulated microgravity can reduce the production of pro-inflammatory cytokines in monocytes and lymphocytes. Taken together, this is the first paper demonstrating an effect of microgravity on resolution-related elements in monocytes.

## 2. Materials and Methods

### 2.1. Chemicals and Reagents

Resolvin D1 (RvD1) (≥95%), Resolvin D1-d5 (RvD1-d5) (≥99%), Resolvin D2 (RvD2) (≥95%), Resolvin D2-d5 (RvD2-d5) (≥95%), Resolvin D3 (RvD3) (≥95%), Resolvin D3-d5 (RvD3-d5) (≥99%), Resolvin D4 (RvD4) (≥95%), Resolvin D5 (RvD5) (≥95%), Resolvin E1 (RvE1) (≥95%), and Resolvin E1-d4 (RvE1-d4) (≥99%) were purchased from Vinci-Biochem (Vinci, FI, Italy); formic acid and acetic acid, LC–MS grade, were from Sigma Aldrich (Dermstadt, Germany); OMIX C18 100 µL cartridges were purchased from Agilent Technologies (Santa Clara, CA, USA); chloroform was purchased from Carlo Erba reagents (Milano, MI, Italy); methanol (MeOH), isopropanol (ISOP), acetonitrile (ACN), chloro-form (CHCl_3_), acetone and water, all UHPLC grade solvent was from VWR (Radnor, PA, USA).

### 2.2. Peripheral Blood Cells Isolation and RCCS-Simulated Microgravity

**Figure 1:**
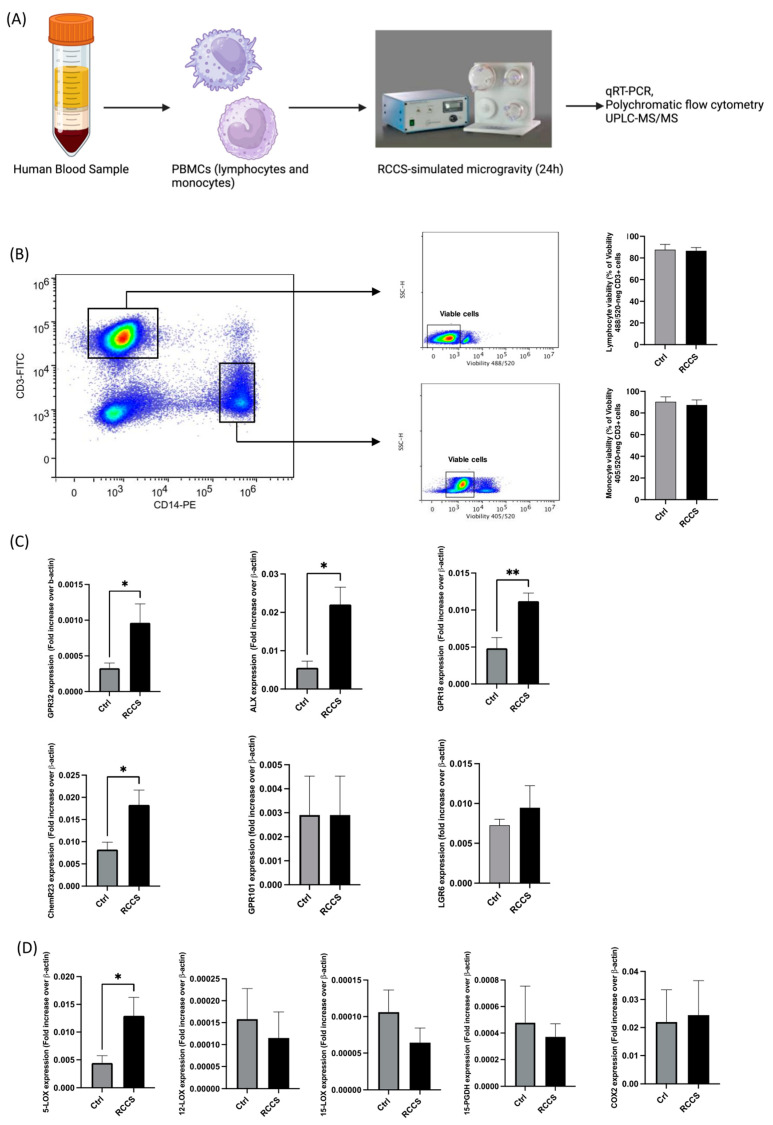
Gene expression of SPM receptors and enzyme in PBMCs cultured for 24 h in RCCS-simulated microgravity. (**A**) Experimental setup: PBMCs were isolated from blood samples of healthy donors by means of gradient density using Ficoll-Hypaque and were kept in complete RPMI for 24 h in a RCCS bioreactor before. Cells were either used immediately for molecular analyses (gene or protein expression assays) or stimulated with LPS or PMA/iono for when the expression of cytokines or biosynthesis of SPMs had to be analyzed. Control samples were kept in the same conditions without undergoing rotation in the RCCS. (**B**) Viability of CD3+ and CD14+ cells after 24 h of RCCS-symulated microgravity: cell viability was assayed by measuring the permeability to Viobility 488/520) (for CD3+ cells) or 405/520 (for CD14+ cells) by flow cytometry. (**C**) qRT-PCR analysis of the genes encoding for the SPM receptors. (**D**) qRT-PCR analysis of the genes encoding for the SPM metabolic enzymes. qRT-PCR data are expressed as 2^(Ctx-CtβA)^ (Ct_x_ is the threshold cycle of each analyzed gene, while Ct_βA_ is the threshold cycle of β-actin, which was used as a normalizing house-keeping gene). All data are shown as mean ± SEM of 6–9 independent experiments, each in duplicate. * *p* < 0.05; ** *p* < 0.01 (paired Student’s *t*-test).

Peripheral blood mononuclear cells (PBMCs) isolated from the buffycoat of healthy donors were separated by density gradient over Lymphosep media (Aurogene) and resuspended at the concentration of 10^6^ cells per ml in RPMI 1640 with 10% fetal bovine serum (FBS), 1% Penicillin/Streptomycin and 1% L-glutamine. Cells were then incubated for 24 h at 1× *g* (Earth gravity) or at ~2 × 10^−3^ g, simulated in the RCCS at a 7.6 rpm rotation, as previously reported [12]. Then, a cell aliquote (5 × 10^5^) was harvested to extract mRNA. Remaining cells were left untreated or were stimulated for 6 h with phorbol-12-myristate-13-acetate (PMA) (50 ng/mL), Ionomicin (1 μg/mL) or for 4 h with lipopolysaccharide (LPS) (100 ng/mL), as previously reported [13]. The experimental setup that was used for the present work is schematized in Figure 1A.

### 2.3. RNA Extraction and qRT-PCR

Total RNA was extracted with the ReliaPrepTM RNA Miniprep Systems (Promega, Madison, WI, USA) according to the manufacture instruction. To synthesize double-stranded cDNA, 200 ng of each RNA sample was reverse transcribed using SensiFAST cDNA Synthesis Kit (Bioline).

Quantitative real-time PCR was performed on ABI PRISM 7900 sequence detector (Applied Biosystems, Waltham, MA, USA) using specific 6-carboxyfluorescein (FAM)-labeled TaqMan assays for: ALX (Hs02759175_s1); ChemR23 (Hs01081979_s1); GPR18 (Hs01921463_s1); GPR32 (Hs00265986_s1); LGR6 (Hs00663887_m1); GPR101 (Hs00369662_s1); 5-LOX (Hs00167536_m1); 12-LOX(Hs00167524_m1); 15-LOX (Hs00993765_g1); 15-PGDH (Hs00960586_g1); and COX-2 (Hs00153133_m1). Analyses were performed on each sample in duplicate, using 5 ng of cDNA per well. In order to calculate the relative fold gene expression of samples was used the 2^−∆Ct^ method, using the β-actin house-keeping gene for normalization.

### 2.4. Flow Cytometry

In order to measure the expression levels of SPM receptors and enzymes in all PBMCs underwent to simulated microgravity, 5 × 10^5^ cells were placed in V-bottom 96-wells plate (Costar Corning, NY, USA) and each well was stained at cell surface with anti-GPR18 (Novusbio Bio-Techne, Minneapolis, MN, USA), anti-GPR32 (Genetex, Irvine, CA, USA), anti-ALX (Miltenyi Biotec, Bergisch Gladbach, Germany), anti-ChemR23 (Miltenyi Biotec) and anti-5-LOX for 30 min at room temperature. Subsequently, a secondary AlexaFluor647-conjugated anti-rabbit secondary antibody was added to all the wells for 25 min in the dark.

In order to identify T cells and monocytes, anti-CD4 (Miltenyi Biotec), anti-CD8 (Miltenyi Biotec) and anti-CD14 (Miltenyi Biotec) antibodies were added to all wells.

In order to measure the intracellular cytokine levels, secretion was inhibited by adding a protein transport inhibitor cocktail 1X (Invitrogen) 5 h before the end of stimulation with either PMA/Ionomycin or LPS (100 ng/mL). At the end of the incubation, cells were stained at cell surface with anti-CD3 (Miltenyi Biotec), anti-CD4 (Miltenyi Biotec) and anti-CD8 (Miltenyi Biotec) for T-cells and anti-CD3 (Miltenyi Biotec) and anti-CD-14 (Miltenyi Biotec) for monocytes at +4 °C for 25 min and fixed in 4% paraformaldehyde at RT for 10 min. Cells were then made permeable with 0.5% saponin and stained intracellularly with anti-TNFα (Miltenyi Biotec), anti-INFγ (Miltenyi Biotec), anti-IL6 (Biolegend, San Diego, CA, USA) and anti-IL12 (Biolegend).

All samples were analyzed using flow cytometry in a CytoFLEX (Beckman Coulter, Miami, FL, USA). For each experimental condition, at least 100,000 events were acquired. The antibodies and the dilution used for flow cytometry detection are listed in Table 1.

### 2.5. UPLC-MS/MS Analysis of SPMs

In order to evaluate the SPMs concentration in PBMC lysates, LC-MS/MS analysis was performed by means of a Aquity UPLC H-Class (Waters, Milford, MA, USA) coupled to Qtrap4500 mass spectrometer (Sciex, Toronto, ON, Canada) according to Fanti et al. 2021 [14] with slight modifications. Briefly, samples were homogenized in 1 mL of MeOH with internal standards (ISs) solution (final concentration 2.5 ng/mL per target analyte) using a Precellys 24 homogenizer (Bertin Technologies, Montigny-le-Bretonneux, France); extractions were then performed on samples by adding 1 mL of H_2_O and 2 mL of CHCl_3_; then samples were centrifuged and the CHCl_3_ portion was removed and dried. Finally, µ-SPE clean-up procedure was performed on dried samples, which were then analyzed using UPLC-MS/MS.

### 2.6. Statistical Analysis

All data are expressed as mean ± SEM. Differences between groups were evaluated by means of parametric (paired *t*-test) or non-parametric (Wilcoxon’s rank test) depending on the fact that normal distribution could be assumed—following the Shapiro–Wilk test—for each experimental group. All statistical tests were performed using PRISM 9.0 (Graphpad Software) and *p*-values < 0.05 were considered significant.

## 3. Results

### 3.1. Simulated Microgravity Rearranges the Expression of Pro-Resolving Genes in PBMCs

The role of microgravity, either simulated or authentic, has never been addressed in the field of resolution of inflammation and of its lipids. At first, we assessed the viability of PBMCs that underwent 24 h incubation in RCCS in our setup by measuring their permeability to Viobility^TM^ (Miltenyi Biotec) using polychromatic flow cytometry and found that vitality was not significantly affected in monocytes nor in lymphocytes by simulated microgravity in our setup (Figure 1B). Then, in order to test the hypothesis that exposure to weightlessness has an effect on the resolution system, we assessed the expression of the genes of known SPM receptors—i.e., GPR32, formyl peptide receptor 2 (ALX, also known as FPR2), GPR18, ChemR23, GPR101 and LGR6—and biosynthetic enzymes—i.e., 5-, 12- and 15-LOX and COX2 [11,15]—in human PBMCs that underwent 24 h of RCCS-simulated microgravity and compared them with control cells incubated under the same conditions as Earth’s gravity. As shown in Figure 1, microgravity enhanced the expression of the majority of the genes encoding for the main SPM receptors, with GPR32 and ALX displaying statistically significant (*p* < 0.05) ~300% and ~400% increases (Figure 1B), respectively, and GPR18 and ChemR23 showing a statistically significant ~200% increase (*p* < 0.01 and *p* < 0.05, respectively) (Figure 1B). Instead, GPR101 and LGR6 were not significantly affected.

As for the effect of simulated microgravity on the gene expression of SPM biosynthetic and degradative enzymes, Figure 1C shows a significant ~300% upregulation (*p* < 0.05) of 5-LOX gene—whose product specifically participates to the synthesis of D-series resolvins—in PBMCs that were cultured for 24 h in RCCS (Figure 1C), while 12- and 15-LOX were downregulated (yet not significantly) by ~20% and ~40%, respectively. The expression of 15-PGDH—i.e., the main enzyme that catalyzes oxidative inactivation of many SPMs—and that of COX-2 were not affected by simulated microgravity (Figure 1C).

### 3.2. Simulated Microgravity Changes Protein Expression of SPM Receptors and Enzymes in Monocytes

**Figure 2:**
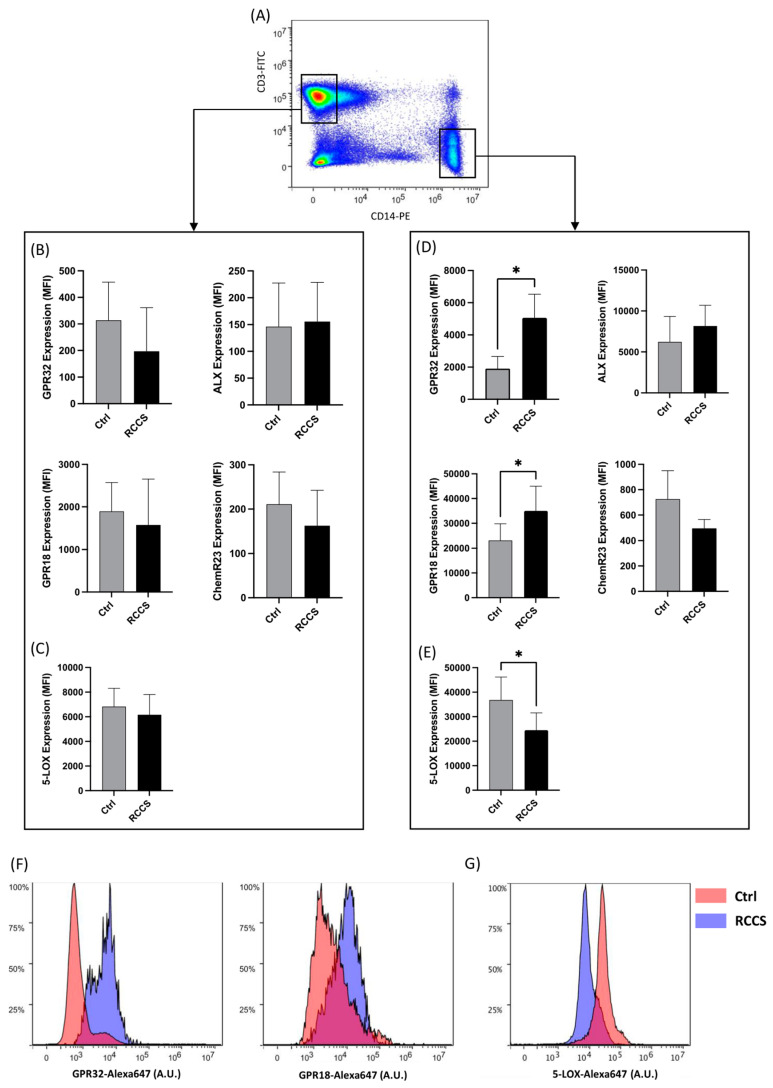
Protein expression of SPM receptors and metabolic enzymes in lymphocytes and monocytes cultured for 24 h in RCCS-simulated microgravity. (**A**) Representative scatter plot showing the gating strategy for polychromatic flow cytometry experiments: T lymphocytes and monocytes were gated as CD3+ and CD14+ cells, respectively, after being appropriately gated according to their viability and physical parameters (1 × 10^6^ cells per condition), before measuring the immunoreactivity of each marker. (**B**–**E**) Expression of GPR32, ALX, GPR18, ChemR23 and 5-LOX in CD3+ T lymphocytes (**B**,**C**) and CD14+ monocytes (**D**,**E**). Data are shown as arbitrary units of mean fluorescence intensity (MFI) of six to nine independent experiments. * *p* < 0.05 (paired Student’s *t*-test). (**F**,**G**) Representative plots showing the distribution of immunofluorescence (polychromatic flow cytometry) for GPR32 and GPR 18 (**F**) and 5-LOX (**G**) in CD14+ cultured at 1 g- and μg.

Given that qRT-PCR was conducted on PBMCs exposed to simulated microgravity, these experiments did not provide specific information on the relative contribution of specific immune cell types on the observed variations. Thus, we sought not only to investigate whether the protein products of genes that were significantly affected by simulated microgravity could be affected as well but also to understand whether these changes are linked to specific immune subsets. Thus, we assayed the differential expression of SPM-related receptors and enzymes in human primary PBMC-derived monocytes and lymphocytes by means of polychromatic flow cytometry. The gating strategy is shown in Figure 2A. Both lymphocytes and monocytes displayed immunoreactivity for GPR32, ALX/FPR2, GPR18 and ChemR23 at 1× *g*, even though their expression was significantly higher in monocytes. In lymphocytes, 24 h exposure to RCCS-simulated microgravity led to a slight—yet not significant—downregulation of GPR32, GPR18 and ChemR23 (Figure 2B), while the expression of ALX/FPR2 remained unchanged. As for monocytes, their GPR32 and GPR18 receptors were significantly upregulated by microgravity (*p* < 0.05) by ~250% and ~50%, respectively (Figure 2D,F).

5-LOX was found to be expressed in both lymphocytes and monocytes, the latter cells exhibiting a significantly higher immunoreactivity (Figure 2C,E), which is consistent with the key role of innate immune cells in the production of LOX-derived lipids (e.g., eicosanoids and SPMs). Interestingly, whereas CD3+ T did not display relevant variations in the expression of 5-LOX upon exposure to microgravity, a significant ~30% downregulation was observed in monocytes under the same conditions, suggesting a cell-specific response to microgravity of SPM metabolic machinery (Figure 2E,G).

### 3.3. SPM Biosynthesis Changes in PBMCs Exposed to Simulated Microgravity

**Figure 3:**
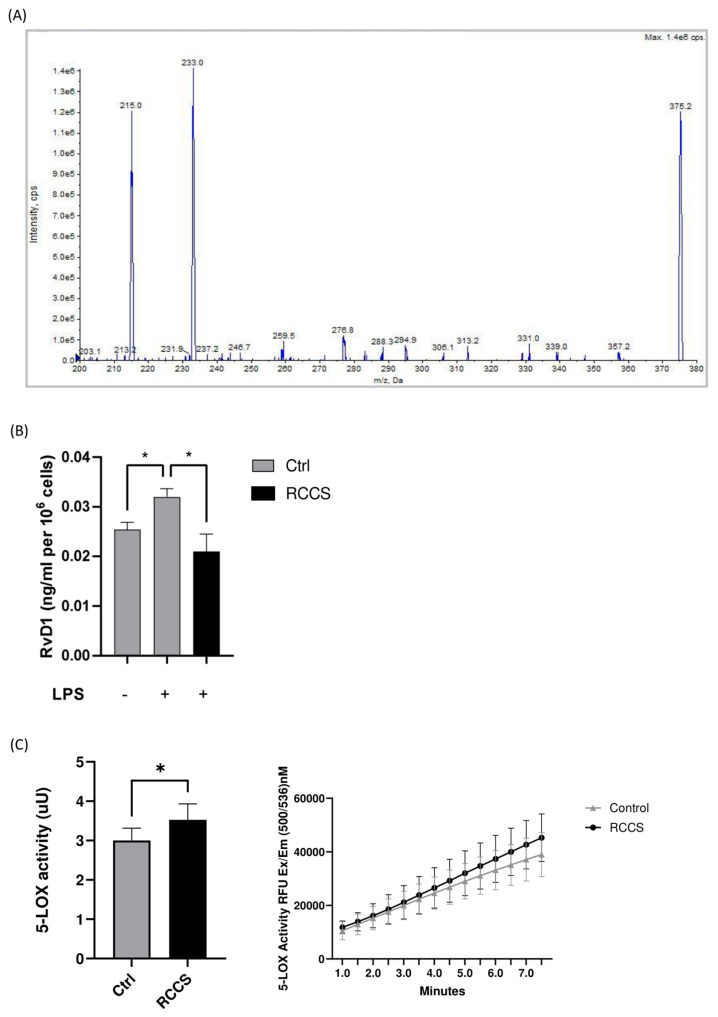
Endogenous levels of RvD1 in LPS-stimulated PBMCs. (**A**) Representative tandem mass spectrum for RvD1. (**B**) Quantitation of RvD1 in PBMCs that were exposed to RCCS-simulated microgravity for 24 h before being stimulated with LPS 100 ng/mL (1 × 10^6^ cells per condition). (**C**) 5-LOX activity. Data are expressed as mean ± SEM of four independent experiments. * *p* < 0.05 (paired *one-way* ANOVA and Bonferroni’s post hoc test).

Given the effect of RCCS-microgravity on GPR32 and GPR18 receptors—which engage two prominent members of D-series resolvins like RvD1 and RvD2—as well as on 5-LOX—which catalyzes the final steps in the production of the same RvDs—we sought to further interrogate whether the biosynthesis of both RvDs might be impaired in microgravity. Thus, we measured RvD1 and RvD2 content in PBMCs that were treated with either vehicle or LPS to specifically stimulate monocytes under simulated weightlessness conditions. Our HPLC-MS/MS data (the MS/MS spectra of detected SPM are showed in Figure 3A) show that LPS stimulation led to a significant ~20% increase in the production of RvD1 in cells cultured at 1× *g*, as well as in microgravity (Figure 3B). Incidentally, the amounts of RvD1 measured in unstimulated cells that underwent microgravity were comparable to those found in 1× *g* cells. Instead, we failed to detect RvD2 in our cells, either in Earth gravity or under microgravity. We also performed an affinity assay on 5-LOX in order to test the possibility that simulated microgravity has an effect on the enzyme ability to catalyze the biosynthesis of SPMs per se and found that the 24 h incubation in RCCS resulted in the slightly enhanced activity of 5-LOX (*p* < 0.05) (Figure 3C).

### 3.4. Simulated Microgravity Modifies Cytokine Production in Both Lymphocytes and Monocytes

**Figure 4:**
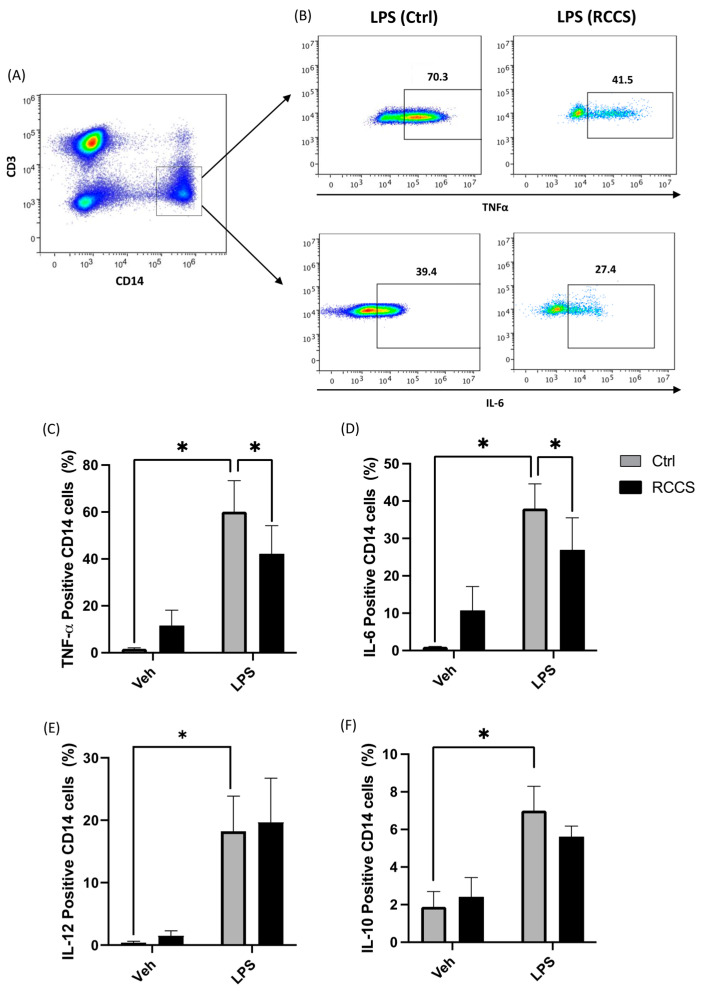
Cytokine production in monocytes cultured for 24 h in RCCS-simulated microgravity. (**A**) Representative scatter plot showing the gating strategy to assess cytokine production: accumulation of intracellular cytokine was assayed in PBMC-derived monocytes by means of polychromatic flow cytometry, and monocytes were gated as CD14+ cells after being appropriately gated according to their viability and physical parameters (1 × 10^6^ cells per condition). (**B**) Representative scatter plots showing the relative production of TNFα and IL-6 in LPS-stimulated monocytes derived from PBMCs that were cultured for 24 h at 1× *g* or RCCS-simulated μg. (**C**–**F**) Intracellular production of TNFa, IL-6, IL-12 and IL-10. Data are shown as means ± SEM of six to nine independent experiments. * *p* < 0.05 (paired *one-way* ANOVA and Bonferroni post hoc test).

The observation that subjects exposed to microgravity display impaired immune reactions to pathogens and altered inflammation suggests that this condition alters the homeostatic properties of immune cells. To date, only scarce data are available on monocytes, though they could be primary targets of microgravity for what concerns the resolution system, as well as pivotal inducers of acute inflammation. Here, we measured the intracellular production of pro-inflammatory cytokines in LPS-stimulated PBMCs via polychromatic flow cytometry and the gating strategy shown in Figure 4A. As expected, 6 h of LPS stimulation triggered a relevant increase in the intracellular production of TNFα, IL-6, IL-12 and IL-10 in monocytes kept at 1× *g*, compared to vehicle-treated samples (Figure 4B–F). Instead, PBMCs kept for 24 h in the RCCS before being stimulated with LPS showed reduced levels of cytokines as compared to 1× *g* controls. In particular, the production of TNFα and IL-6 underwent a significant reduction (*p* < 0.05) down to ~60% and 70% (Figure 4B–D), in respect with control samples, while IL-12 was not affected by simulated microgravity (Figure 4E). Production of IL-10, on the other hand, was only slightly decreased by exposure to simulated microgravity, although this change did not reach statistical significance (Figure 4E). Incidentally, controls exposed to microgravity without LPS stimulation showed an increase in the basal production of the aforementioned cytokines, which, however, was not statistically significant (Figure 4C–E).

**Figure 5:**
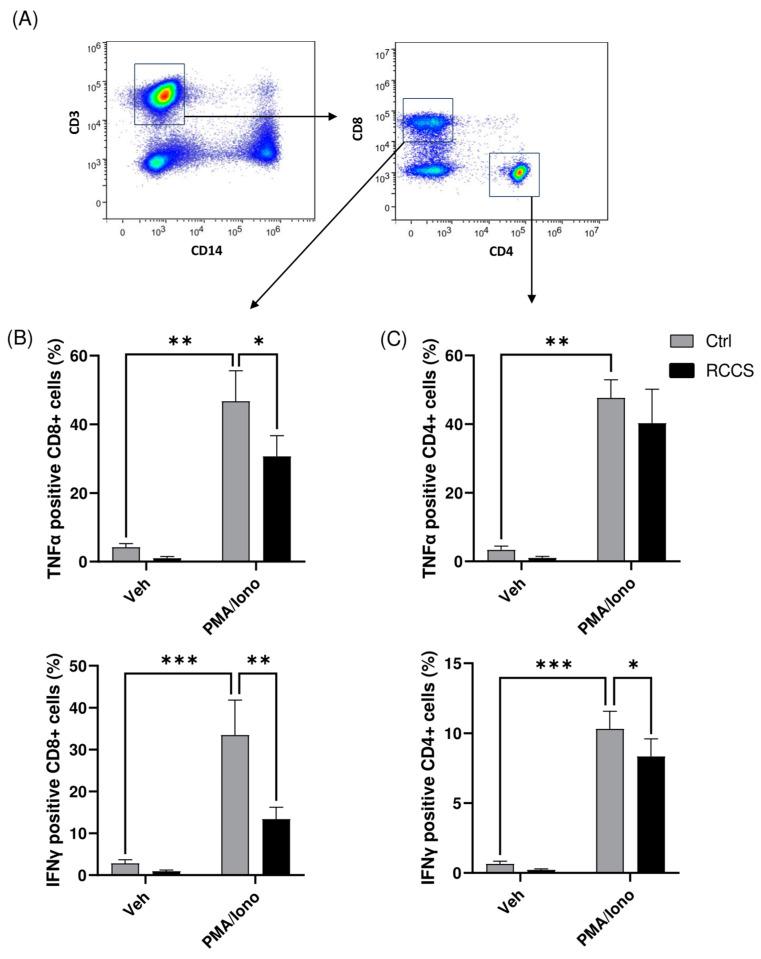
Cytokine production in CD4+ and CD8 T cells. (**A**) Representative scatter plots showing the gating strategy to identify CD4 and CD8 T cells (**B**,**C**) Intracellular production of TNFa and IFNg in CD4 and CD8 cells assayed using polychromatic flow cytometry. Data are shown as means ± SEM of six to nine independent experiments. * *p* < 0.05; ** *p* < 0.01; *** *p* < 0.001 (paired *one-way* ANOVA and Bonferroni’s post hoc test).

We also analyzed the production of lymphocyte-associated cytokines in the same samples, as shown in Figure 5, and found a slight decrease in the production of TNFα and a ~20% reduction (*p* < 0.05) in that of IFNγ in CD4+ cells (Figure 5B,C). By comparison, the effect on CD8+ cells was much more evident, with microgravity causing a significant drop in the production of TNFα and IFNγ of ~25% (*p* < 0.05) and ~40% (*p* < 0.01) (Figure 5).

## 4. Discussion

An altered or ineffective resolution program is often associated with immune and inflammatory [11,16]. Although microgravity has long been associated with modifications of the immune network that lead to enhanced phlogosis or impaired clearance of pathogens, its effect on the resolution network has never been addressed. SPMs are orchestrators of the resolution process, and they participate to the active control of the inflammatory surge that is either triggered by infective events or by other exogenous pathogenic stressors [10,11,15]. To date, an impairment of the resolution machinery, or its inability to counteract uncontrolled inflammatory signals, has been linked to a number of known pathologies [11] and is thought to be involved in virtually all phlogistic disorders. However, despite their emerging role in immune control—and the previously documented effect of microgravity on other cognate immunomodulatory lipids (e.g., AA-derived eicosanoids and endocannabinoids) [4]—SPMs still represent an unexplored territory in space biomedical research. Here, we show the effect of simulated weightlessness on key resolution receptors, as well as on the production of pro-resolving mediators. Indeed, RCCS-simulated microgravity significantly changes the expression fingerprint of pivotal resolution elements in PBMCs, upregulating the genes encoding for GPR32 and ALX (that are RvD1 and LXA_4_ receptors), GPR18 (a RvD2 receptor) and ChemR23 (a RvE1 receptor), as well as the gene encoding for 5-LOX, which controls the biosynthesis of crucial D-series resolvins, including RvD1. Interestingly, the effect of microgravity on GPR32 and GPR18 overexpression was specific to monocytes and could not be demonstrated in lymphocytes under the same experimental conditions. Remarkably, monocytes represent crucial players in inflammation and in resolution and are important targets of SPMs that show a reduced effect on them during inflammatory diseases [17,18].

Interestingly, the microgravity-induced upregulation of 5-LOX gene—which might counterbalance the observed downregulation of the protein—reinforces the concept that this enzyme is crucial for human cell adaptation to microgravity. Indeed, we have previously reported that 5-LOX is responsible for the apoptosis and reduced proliferation of PBMCs under both simulated and authentic microgravity onboard the International Space Station [12,19]. Moreover, we have demonstrated that parabolic flight-induced microgravity enhances substrate affinity (Km) of a purified 15-LOX form, speaking in favor of this enzyme family as “gravity sensor” [20]. These past data, alongside the fact that we observed an increase in the activity of 5-LOX in samples exposed to simulated microgravity, suggest that LOX isozymes might work together as gravity sensors, which upon responding to weightlessness might have an impact on the metabolism of many bioactive lipids. On the other hand, the reduced ability of RCCS-cultured monocytes to produce RvD1 compared to 1× *g* samples shown here is fully coherent with the concomitant downregulation of 5-LOX in the same cells, where the upregulation of SPM receptors might possibly reflect a compensatory attempt. In this context, the intense downregulation of the 5-LOX protein levels might overcome its gravity-induced increased activity, which would lead, in turn, to RvD1-impaired biosynthesis.

Monocytes and macrophages have been previously reported to initiate pro-resolving signals (i.e., the production of SPMs or the upregulation of SPM-binding receptors) upon exposure to Toll-like receptor (TLR) ligands of bacterial origin (i.e., LPS) or to pro-inflammatory cytokines [17,21]. Since microgravity has been shown to blunt the effects of TLR- and of cytokine-dependent signaling in monocytes and macrophages and to hinder their differentiation, activation and polarization [22,23], our data suggest that this effect of microgravity also engages monocytes and their ability to produce SPMs upon the activation of microgravity-sensible Toll signals. Interestingly, the blunting effect that microgravity elicits on both pro-inflammatory (i.e., TNFα, IL-6 and IL-12) and anti-inflammatory cytokines (i.e., IL-10) produced by monocytes suggests that this stressor acts by generally depressing immune functions in these cells, rather than exerting a differential effect on soluble mediators.

Finally, our data show that RCCS-simulated microgravity has a depressing effect on cytokine production from T lymphocytes (both CD4+ and CD8+ cells) and monocytes, which is in keeping with previous reports [23,24,25,26]. On the other hand, other works have reported that immune cells of different origin display a different behavior, when exposed to microgravity: indeed, the splenocytes of mice that experienced spaceflight feature upregulation of proinflammatory (e.g., IFNγ and MIP-1α) and anti-inflammatory mediators (e.g., IL-10), while other mediators, such as IL-2 or TNFα, are reduced [27,28].

Interestingly, lymphocytes have represented a primary target of investigation since the first pioneering studies of space biomedicine; instead, the studies on the innate branch of the immune network are much less abundant—especially for monocytes—with most efforts focusing on monocyte-derived macrophages and, in some cases, on monocyte/macrophage-derived cytokines that are assayed in the blood of animal models or astronauts that experienced weightlessness [29], rather than on circulating monocytes per se.

## 5. Conclusions

The present study reports unprecedented cell-specific effects of microgravity on the pro-resolving machinery that orchestrates SPM effects. In particular, we show that 24 h of RCCS-simulated microgravity can modify the gene and protein expression of SPM receptors and enzymes, leading to reduced endogenous levels of RvD1, a prominent member of DHA-derived resolvins that has been associated with several inflammatory pathologies, and used to attenuate their clinical phenotype [11]. Of note, reduced RvD1 is consistent with the reduced expression of 5-LOX, an enzyme which controls its biosynthesis.

Previous literature focused on the microgravity-caused alterations of the inflammatory network that result in dysfunctional immune processes and consequent diseases. However, tissue homeostasis is the result of an intricate process whereby SPMs work in concert with other lipid messengers to avoid irreversible cell damage. To date, no previous reports have investigated the effect of authentic or simulated weightlessness on bona fide pro-resolving signals. Our data not only further support the prevalent idea that microgravity disrupts immune functions in both monocytes and lymphocytes but it also demonstrates that this effect extends on pro-resolving processes by reducing the ability of monocytes to produce—or respond to—SPMs in inflammatory contexts such as those represented by the agonism of TLR receptors. These data represent an important starting point in lipid space biology to further our understanding of the molecular pathogenesis of diseases associated with space travel-related stressors and to design possible preemptive therapies that potentiate the pro-resolving network.

## Figures and Tables

**Table 1:** Antibodies used for immunostaining in polychromatic flow cytometry experiments.

Antibody	Ab-Conjugated Fluorophore	Manufacturer	Dilution
CD3	FITC	Miltenyi Biotec, Bergisch Gladbach, Germany	1:80
CD4	APC-Vio770	Miltenyi Biotec, Bergisch Gladbach, Germany	1:50
CD8	VioGreen	Miltenyi Biotec, Bergisch Gladbach, Germany	1:50
CD14	APC	Miltenyi Biotec, Bergisch Gladbach, Germany	1:50
GPR-18	Non-conjugated	Novus Bio, Minneapolis, MI, USA	1:200
GPR-32	Non-conjugated	Genetex, Irvine, CA, USA	1:200
5-LOX	Non-conjugated	Abcam, Cambridge, UK	1:100
ALX	APC	Miltenyi Biotec, Bergisch Gladbach, Germany	1:50
ChemR23	APC	Miltenyi Biotec, Bergisch Gladbach, Germany	1:50
TNF-α	PE	Miltenyi Biotec, Bergisch Gladbach, Germany	1:80
IFN- γ	Vioblue	Miltenyi Biotec, Bergisch Gladbach, Germany	1:50
IL-6	PE CF594	Biolegend, San Diego, CA, USA	1:400
IL-10	PE	BD Biosciences, San Jose, CA, USA	1:50
IL-12	BV421	Biolegend, San Diego, CA, USA	1:400
IL-17	PE-Vio770	Miltenyi Biotec, Bergisch Gladbach, Germany	1:50
Goat anti-Rabbit	Alexa Fluor 647	Southern Biotech, Birmingham, AL, USA	1:200

## Data Availability

None of the data supporting the findings of this study were derived from public repositories or databases.
